# *SMAD6* variants in craniosynostosis: genotype and phenotype evaluation

**DOI:** 10.1038/s41436-020-0817-2

**Published:** 2020-06-05

**Authors:** Eduardo Calpena, Araceli Cuellar, Krithi Bala, Sigrid M. A. Swagemakers, Nils Koelling, Simon J. McGowan, Julie M. Phipps, Meena Balasubramanian, Michael L. Cunningham, Sofia Douzgou, Wanda Lattanzi, Jenny E. V. Morton, Deborah Shears, Astrid Weber, Louise C. Wilson, Helen Lord, Tracy Lester, David Johnson, Steven A. Wall, Stephen R. F. Twigg, Irene M. J. Mathijssen, Freya Boardman-Pretty, Genomics England Research Consortium, F. Boardman-Pretty, Simeon A. Boyadjiev, Andrew O. M. Wilkie

**Affiliations:** 1 MRC Weatherall Institute of Molecular Medicine, University of Oxford, John Radcliffe Hospital, Oxford, UK; 2 Department of Pediatrics, University of California–Davis, Sacramento, CA USA; 3 Departments of Pathology and Bioinformatics, Erasmus MC, University Medical Center Rotterdam, Rotterdam, The Netherlands; 4 Sheffield Clinical Genetics Service, Sheffield Children’s NHS Foundation Trust, Sheffield, UK; 5 Division of Craniofacial Medicine, Department of Pediatrics, University of Washington, Seattle, WA USA; 6 Manchester Centre for Genomic Medicine, Central Manchester University Hospitals NHS Foundation Trust, Saint Mary’s Hospital, Manchester, UK; 7 Division of Evolution and Genomic Sciences, School of Biological Sciences, Faculty of Biology, Medicines and Health, University of Manchester, Manchester, UK; 8 Fondazione Policlinico Universitario A. Gemelli IRCCS, Rome, Italy; 9 Department of Life Science and Public Health, Università Cattolica del Sacro Cuore, Rome, Italy; 10 West Midlands Regional Clinical Genetics Service and Birmingham Health Partners, Birmingham Women’s and Children’s Hospitals NHS Foundation Trust, Birmingham, UK; 11 Oxford Centre for Genomic Medicine, Oxford University Hospitals NHS Foundation Trust, Oxford, UK; 12 Craniofacial Unit, Oxford University Hospitals NHS Trust, John Radcliffe Hospital, Oxford, UK; 13 Department of Clinical Genetics, Liverpool Women’s NHS Foundation Trust, Liverpool, UK; 14 Clinical Genetics Service, Great Ormond Street Hospital for Children NHS Foundation Trust, London, UK; 15 Oxford Genetics Laboratories, Oxford University Hospitals NHS Foundation Trust, The Churchill Hospital, Oxford, UK; 16 Department of Plastic and Reconstructive Surgery and Hand Surgery, Erasmus MC, University Medical Center Rotterdam, Rotterdam, the Netherlands; 17 Genomics England, London, UK; 18 William Harvey Research Institute, Queen Mary University of London, London, UK

**Keywords:** BMP2, metopic synostosis, digenic inheritance, two-locus, protein instability

## Abstract

**Purpose:**

Enrichment of heterozygous missense and truncating *SMAD6* variants was previously reported in nonsyndromic sagittal and metopic synostosis, and interaction of *SMAD6* variants with a common polymorphism near *BMP2* (rs1884302) was proposed to contribute to inconsistent penetrance. We determined the occurrence of *SMAD6* variants in all types of craniosynostosis, evaluated the impact of different missense variants on SMAD6 function, and tested independently whether rs1884302 genotype significantly modifies the phenotype.

**Methods:**

We performed resequencing of *SMAD6* in 795 unsolved patients with any type of craniosynostosis and genotyped rs1884302 in *SMAD6*-positive individuals and relatives. We examined the inhibitory activity and stability of SMAD6 missense variants.

**Results:**

We found 18 (2.3%) different rare damaging *SMAD6* variants, with the highest prevalence in metopic synostosis (5.8%) and an 18.3-fold enrichment of loss-of-function variants comparedwith gnomAD data (*P* < 10^−7^). Combined with eight additional variants, ≥20/26 were transmitted from an unaffected parent but rs1884302 genotype did not predict phenotype.

**Conclusion:**

Pathogenic *SMAD6* variants substantially increase the risk of both nonsyndromic and syndromic presentations of craniosynostosis, especially metopic synostosis. Functional analysis is important to evaluate missense variants. Genotyping of rs1884302 is not clinically useful. Mechanisms to explain the remarkable diversity of phenotypes associated with *SMAD6* variants remain obscure.

## INTRODUCTION

Craniosynostosis (CRS), the premature fusion of the cranial sutures, is a heterogeneous disorder with a prevalence of ∼1 in 2000. Environmental factors, polygenic inheritance and single-gene or chromosomal abnormalities all contribute to its complex manifestations. Variants in >60 genes have been identified as recurrently associated with CRS, with an underlying genetic cause being found in ∼24% of patients overall.^1–3^ The proportion in whom a cause can be determined varies widely depending on clinical diagnosis: from 88% for bicoronal synostosis down to 8% for sagittal synostosis (SS).^2^ Until recently, success in identifying a genetic diagnosis has been particularly low in nonsyndromic midline CRS, under 1% for both sagittal and metopic suture fusions.^2^

In 2016, Timberlake et al.^4^ performed exome sequencing of 132 parent–offspring trios and 59 additional probands presenting with clinically nonsyndromic SS, metopic (MS), or combined metopic/sagittal synostosis, seeking evidence for major monogenic contributions to these disorders. Based on enrichment of de novo variants and inherited damaging variants, this study identified a single significant gene, *SMAD6*, located at 15q22.3.^4^

*SMAD6*, originally identified in mammals by homology-based cloning,^5,6^ encodes one of two (with SMAD7) inhibitory members of the SMAD family required for regulated intracellular signal transduction by members of the transforming growth factor β/bone morphogenetic protein (TGFβ/BMP) superfamily.^7–9^ Intriguingly, enrichment of rare *SMAD6* variants has also been reported in association with several other distinct phenotypes, namely congenital heart disease,^10–12^ bicuspid aortic valve (BAV) and ascending thoracic aortic aneurysm (TAA),^13–15^ intellectual disability,^16^ and radioulnar synostosis.^17^

In a follow-up study, Timberlake et al. increased the sample size of probands with midline CRS and no other genetic diagnosis to 379 (45 pedigrees included ≥1 additional affected family member).^18^ They found damaging *SMAD6* variants in 4/234 (1.7%) SS, 11/135 (8.1%) MS, and 2/10 (20%) combined metopic/sagittal synostosis probands. Although de novo variants (DNMs) were identified in four families, in the remainder, the *SMAD6* variant was transmitted by an apparently unaffected (i.e., nonpenetrant) parent. Similar observations of nonpenetrance of *SMAD6* variants were made for several of the other described disease associations.^13,14,17^ To seek an explanation for the unpredictable penetrance, Timberlake et al.^4,18^ genotyped a single-nucleotide polymorphism (SNP), rs1884302, previously reported in a genome-wide association study (GWAS) of nonsyndromic SS to be the most significant associated SNP, which may differentially regulate the most proximal gene *BMP2*.^19,20^ The risk-conferring C allele (prevalence in non-Finnish Europeans of 32.7%, gnomAD),^21^ was found to be present in 15/21 individuals with CRS but only 1/20 unaffected relatives heterozygous for the *SMAD6* variant but without CRS, suggesting a two-locus mechanism to account for variable manifestation of CRS.^18^

Although the studies described above^4,18^ represent an important advance in delineating the contribution of single-gene variants to nonsyndromic midline CRS, they raise several questions. First, what is the contribution of *SMAD6* variants in all presentations of CRS (including syndromic diagnoses and fusion of coronal or lambdoid sutures)? Second, can it be assumed that all rare SMAD6 missense variants affect protein function? Third, can the two-locus (*SMAD6*/rs1884302) model be confirmed in an independent cohort? Here, we address these questions. We confirm the primary finding that *SMAD6* variants are enriched in CRS, especially metopic synostosis, but find a more diverse pattern of clinical presentation; in addition, we illustrate the importance of combining functional studies with frequency-based evaluation of variants to refine likelihood of pathogenicity. Finally, we report that the two-locus model does not account for inconsistencies of penetrance of damaging *SMAD6* variants in our data set.

## MATERIALS AND METHODS

### Patients

The clinical studies were approved by respective Institutional Review Boards (IRB): Oxfordshire Research Ethics Committee (REC) B (C02.143), London–Riverside REC (09/H0706/20), East of England–Cambridge South REC (14/EE/1112 for 100,000 Genomes Project [100kGP]), the Medical Ethical Committee of the Erasmus University Medical Center Rotterdam (MEC-2012–140), and the IRB of the University of California–Davis (International Craniosynostosis Consortium [ICC]; protocol 215635–23). Written informed consent to obtain samples for genetics research was given by each child’s parent or guardian. Written authorization for publication of clinical photographs was obtained from every individual and/or their parent/guardian. The clinical diagnosis of CRS was confirmed by three-dimensional computed tomography scanning of the skull; routine genetic investigations are described in Supplementary Information. Patients were considered to have a syndromic diagnosis if (1) additional dysmorphic features or congenital anomalies were present and/or (2) there was significant developmental delay or intellectual disability on neuropsychological assessment; in addition (3) families including affected first degree relatives were classified as syndromic, although affected individuals in those families may have presented with nonsyndromic clinical features.

### Resequencing, bioinformatics, variant validation, and dosage analysis

Variant screening was performed by next-generation sequencing (NGS)-based resequencing of polymerase chain reaction (PCR) products encompassing the coding regions and intron/exon boundaries of the four exons of *SMAD6* (NC_000015.10 [chr15:66702110–66782849, hg38]; NM_005585, ENST00000288840.9). Primer sequences are given in Table S1 and detailed methods are provided in Supplementary Information. Variant calls and coverage information were obtained using the bioinformatic tool amplimap.^22^

Validation and segregation analysis of variants was undertaken by dideoxy-sequencing of PCR products from genomic DNA. The rs1884302 *BMP2* polymorphism was genotyped in all the *SMAD6*-positive individuals by PCR (Table S1) followed by EcoRI digest and/or dideoxy-sequencing.

Analysis of *SMAD6* dosage using multiplex ligation-dependent probe amplification (MLPA) is described in the Supplementary Information.

### Frequency- and deleteriousness-based variant stratification

Using the frequency-based filtering framework provided by Whiffin et al.,^23^ we estimated the maximum credible population allele frequency (AF) for any causative variant in CRS to be 0.000045, assuming a penetrance of 0.2 (Supplementary Information). For each identified variant we assigned AF_max_, the greater of (1) the maximum observed AF in any population in gnomAD^21^ (except Other; gnomAD v2.1.1), where this was based on the presence of ≥2 mutant alleles in that population, or (2) the overall AF. We classified rare alleles as those for which AF_max_ was <0.000045.

To measure the deleteriousness of the identified variants, the deleterious score (DS, range 0–6) was calculated based on exceeding a defined threshold for six separate scores generated by Annovar^24^ (Supplementary Information).^25^ For comparison, the CADD score was additionally calculated for missense variants.^26^

For all *SMAD6* variants listed in previous publications^4,10–18^ and implied to be pathogenic, nomenclature was verified using Mutalyzer^27^ and their presence and AF checked in gnomAD. We calculated the DS and CADD score for all missense variants.

### Functional analysis

Source plasmids and methods used to evaluate the 5′ untranslated region (UTR), splice-site, and missense variants are provided in the Supplementary Information. Luciferase measurements and immunoblot quantifications were performed from at least three independent experiments. Statistical analysis is described in the Supplementary Information.

## RESULTS

### Resequencing of *SMAD6* in 795 patients with CRS of unknown etiology

**Fig. 1:**
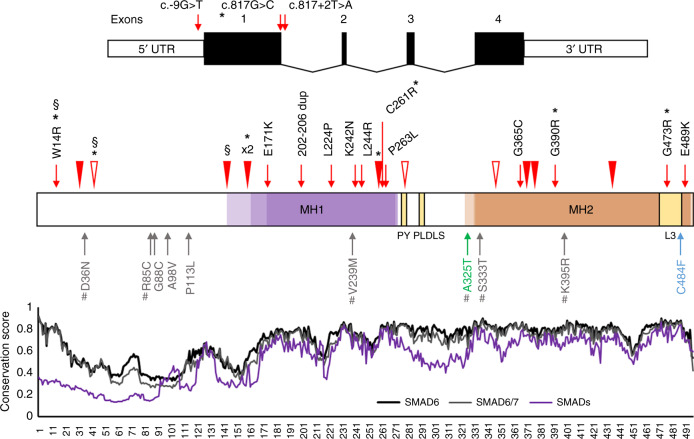
Human *SMAD6* gene and protein showing variants identified in craniosynostosis (CRS). Top, *SMAD6* comprises four exons; positions of variants affecting translation initiation or splicing are indicated. Middle, cartoon of encoded protein showing conserved domains (MH1 and MH2, including highly conserved L3 region) and PY and PLDLS motifs.^9^ Colored shading indicates the position of the MH1 (transparent purple) and MH2 (transparent orange) domains according to Uniprot, Pfam, and CDD resources, with darker shading denoting overlapping domain assignments. Novel or rare (AF_max_ < 0.000045) variants identified in CRS patients that are also predicted damaging (loss-of-function [LoF], plus missense variants with DS^25^ ≥ 4) are indicated above the cartoon, whereas below in gray are additional missense variants predicted to have lower pathogenicity (DS ≤ 3 and/or AF_max_ > 0.000045); negative and positive controls^10^ used in the functional assays are colored green and blue, respectively. Frameshifts and stop-gain variants are shown with filled and empty arrowheads, respectively; § = de novo variants; * = novel/rare damaging variants found in addition to the CRS cohort screen; # = AF_max_ ≥ 0.000045 in gnomAD. Bottom, conservation profiles^40^ of inhibitory SMADs SMAD6 (black), SMAD7 (gray), and all SMAD members (SMAD1–8) combined (purple line). *AF* allele frequency, *DS* deleterious score.

**Fig. 2:**
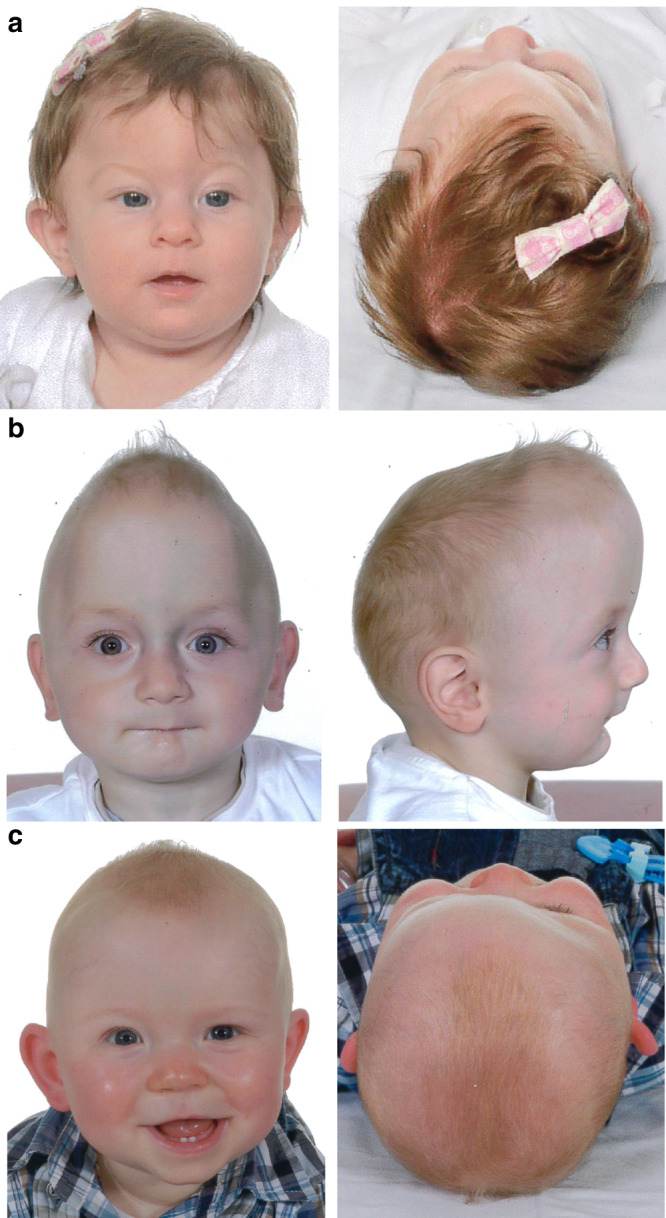
Preoperative clinical presentations of craniosynostosis (CRS) in association with pathogenic heterozygous *SMAD6* variants. (**a**) Subject 8260 aged 4 months with metopic synostosis, the most frequently associated CRS phenotype, showing hypotelorism (front view) and trigonocephaly (top view). Newly described clinical presentations include sagittal and bicoronal synostosis (**b**, subject 3711 aged 11 months, note narrow, saddle-shaped skull with frontal bossing) and right unicoronal synostosis (**c**, subject 4370 aged 10 months, note facial asymmetry and recessed brow on right).

To investigate the contribution of *SMAD6* variants in CRS, we performed NGS-based resequencing of *SMAD6* in 795 unsolved patients with any type of CRS. After applying the joint criteria that variants should be both rare (AF_max_ < 0.000045) and damaging (predicted loss-of-function [LoF] and/or DS ≥ 4/6), we identified 18 probands (and 2 additional affected siblings) with heterozygous rare damaging *SMAD6* variants (Table 1, Fig. 1, Fig. S1, Table S2). These *SMAD6*-positive individuals accounted for 2.3% of the cohort (3.4% and 2.0% of those classified as syndromic and nonsyndromic, respectively). Nine probands had LoF variants, representing a ~18.3-fold enrichment compared with gnomAD genome sequencing data (9 LoF in minimum 29,066 alleles; *P* < 10^−7^, Fisher’s exact test). The highest prevalence of novel/rare damaging *SMAD6* variants occurred in MS (12/207; 5.8%). Such *SMAD6* variants were much less frequent in isolated SS (3/316; 0.95%) or other types of suture fusion, but we noted three probands with coronal suture involvement (one each sagittal + bicoronal, sagittal + unicoronal, and unicoronal synostosis). The varied craniofacial presentation of individuals with CRS heterozygous for *SMAD6* variants is illustrated in Fig. 2a–c.

**Table 1:** Subjects with CRS analyzed for rare, deleterious *SMAD6* variants by NGS-based resequencing.

	Nonsyndromic		Syndromic		Combined	
	Total	*SMAD6* positive	Total	*SMAD6* positive	Total	*SMAD6* positive
Metopic	167	9	40	3^b^	207	12 (5.80%)
Sagittal	279	2^a^	37	1^b^	316	3 (0.95%)
Unilateral coronal	150	1	16	0	166	1 (0.60%)
Bilateral coronal	11	0	11	0	22	0
Uni- or bilateral lambdoid	7	0	3	0	10	0
Multisuture	35	1	29	1	64	2 (3.13%)
Sutures not specified	0	0	10	0	10	0
Combined	649	13	146	5	795	18 (2.26%)

*CRS* craniosynostosis, *NGS* next-generation sequencing.

^a^In one patient, additional bicoronal suture fusion was noted at the time of surgery.

^b^Includes proband classified as syndromic because a sibling had sagittal synostosis.

Together, these observations confirm the enrichment of rare, damaging *SMAD6* variants in CRS, as reported in nonsyndromic midline synostosis.^4,18^ However, this work extends the previous findings to include syndromic as well as nonsyndromic patients, and synostosis of coronal sutures. Among the pure midline synostoses, MS was 6.1-fold more frequent than SS (*P* = 0.002, two-tailed Fisher’s exact test).

### Phenotypic characterization of *SMAD6*-positive individuals

Combining these unbiased findings with eight additional independently identified (see Supplementary Information) *SMAD6*-positive CRS patients (three MS, three SS, one sagittal + bicoronal and one unicoronal synostosis; Fig. S2), in total we identified 25 different damaging variants in *SMAD6* (Fig. 1, Table S2) in 28 affected individuals from 26 unrelated families with CRS.

The phenotypes identified in each of the 28 *SMAD6*-positive individuals are summarized in Table S3. Of 8 patients considered to have a syndromic clinical presentation, 1 (subject 5944) was excluded from further consideration owing to confounding by two additional DNMs likely to contribute to the phenotype, which had been separately identified by exome sequencing.^28^ Of the remaining seven syndromic subjects, five had congenital heart defects (comprising atrioventricular septal defect, atrial septal defect [ASD] with patent foramen ovale, two ASD with ventricular septal defect, and bicuspid aortic valve with right bundle branch block; only the first of these required corrective surgery), three had brain anomalies (comprising ventriculomegaly and absent corpus callosum, macrocephaly, and mild microcephaly), and one had duodenal atresia. Seven children had delayed developmental/intellectual or educational attainment, classified as mild–moderate in one case and mild in the remainder. Both congenital heart defects^10–12^ and neurodevelopmental disability^16^ were previously described as significantly associated with *SMAD6* variants.

Although the craniofacial surgical course for most patients with CRS was good, four individuals (plus the child with additional confounding DNMs) were documented to have raised intracranial pressure (ICP). Of those four, one with syndromic SS developed additional bilateral coronal suture fusion and was found to have primary raised ICP and the other three (two SS, one MS) had raised ICP following reconstructive craniofacial surgery, necessitating a second major surgical procedure. Secondarily raised ICP represents an infrequent complication of simple synostosis of midline sutures, for example only 2 of 128 patients with SS,^29^ and 6 of 202 patients with MS^30^ who underwent major calvarial remodeling procedures developed secondarily raised ICP. The difference from background is significant (*P* = 0.042, Fisher’s exact test), suggesting that this is a complication that should be monitored for in *SMAD6*-positive CRS.

### Mutational spectrum of *SMAD6* and functional evaluation of the variants

The human SMAD6 protein comprises 496 amino acids and includes two highly conserved domains (MH1 and MH2), with MH2 being necessary and sufficient for its inhibitory activity on TGFβ/BMP signaling.^31,32^ The 25 different rare damaging variants in *SMAD6* include 12 LoF (seven frameshift, three stop-gain, two splice-site), one within the 5′ UTR (creating an out-of-frame upstream ATG), an in-frame duplication (within the MH1 domain) and 11 missense variants (Fig. 1). Ten of the 11 identified missense variants are clustered in the MH1/MH2 domains and the remaining substitution is a de novo p.W14R located near the N-terminus and affecting a highly conserved region of the inhibitory SMADs (SMAD6/SMAD7; Fig. 1). We performed in vitro studies of the *SMAD6* variants (excluding the frameshifts and stop-gains) to gather additional evidence for pathogenicity.

The two splice-site variants (c.817G>C and c.817+2T>A) are predicted to affect recognition of the intron 1 splice donor; in both cases, analysis of messenger RNA (mRNA) extracted from patient cells showed abnormal splice product(s), absent in the control (Fig. S3). Dideoxy-sequencing demonstrated activation of cryptic donor splice sites within intron 1, resulting in partial intron retention and generating a frameshift and premature stop codon (Fig. S3).

The 5′ UTR variant (c.-9G>T), present in both affected individuals of family M130 and assumed to have been inherited from their father (Fig. S1), creates an out-of-frame upstream ATG codon with potential initiation activity. Using a dual-luciferase reporter construct in C2C12 cells, the translation efficiency of the main open reading frame (ORF) was reduced, an effect not observed when the c.-9G>A SNP (rs559095945; AF[total]=0.0001706), affecting the same nucleotide but not generating an ATG codon, was tested (Fig. S4).

**Fig. 3:**
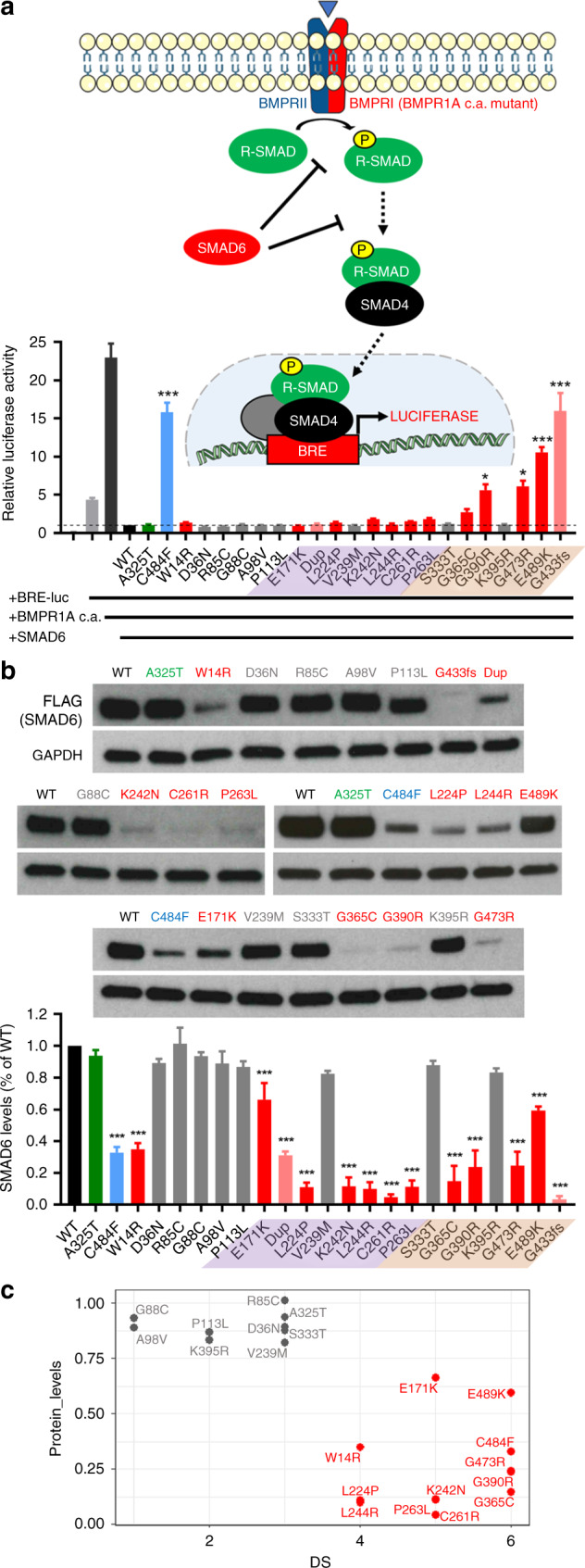
Functional analysis of SMAD6 variants. (**a**) Luciferase assay. The cartoon at top shows a simplified representation of the BMP signaling pathway, indicating in red the components transfected into C2C12 cells to perform the assay. Firefly luciferase activity of the BRE-luc transcriptional reporter induced by constitutively active BMPR1A (BMPR1A c.a.) was used to monitor the inhibitory effects of SMAD6 variants on BMP signaling, similar to previously described.^10^ Below, graphs represent means ± SEM from three independent experiments. Data were normalized (using *Renilla* levels), relativized to the wild type (WT) and analyzed by one-way analysis of variance (ANOVA) with Dunnett’s multiple-comparisons test; ^∗^*P* ≤ 0.05, ^∗∗^*P* ≤ 0.01, and ^∗∗∗^*P* ≤ 0.001. Color-coding of SMAD6 variants follows the same scheme as in Fig. 1. (**b**) Analysis of SMAD6 protein stability. Above are representative examples of western blots (using aliquots of protein extracts from the luciferase assays), showing SMAD6 protein levels (detected with anti-FLAG) compared with anti-GAPDH loading control. Control missense variants (negative, green bar, p.A325T; positive, blue bar, p.C484F) were selected as previously described.^10^ Data were normalized (using GAPDH) and relativized to the WT. The bars represent means ± SEM from three independent experiments, analyzed and visualized as in (**a**). (**c**) SMAD6 average protein levels of 21 different missense variants (from **b**) plotted against respective deleterious score (DS).

The inhibitory activity of the SMAD6 missense variants on BMP signaling was evaluated using a BRE-luc transcriptional reporter containing a BMP-responsive element.^10,33,34^ In addition to the 11 damaging and rare missense variants summarized in Table S2, we included 10 other missense variants that were either previously reported as positive (p.C484F) or negative (p.A325T) controls,^10^ or that we had encountered during resequencing (Table S2) but were classed as being of uncertain pathogenicity because they failed one or both criteria of being damaging and rare (Supplementary Information). SMAD6 missense variants located outside the MH2 domain maintained potent inhibitory activity, similar to the wild type (WT) and the p.A325T negative control (Fig. 3a). For SMAD6 variants located within the MH2 domain, the rare damaging variants associated with CRS (p.G365C, p.G390R, p.G473R, and p.E489K) showed reduced inhibitory capacity (statistically significant except for p.G365C), whereas more frequent and/or less damaging variants (p.S333T, p.K395R) did not. We found a stronger defect in variants within, or near to, the L3 loop motif, indispensable for the receptor association (Figs. 1 and 3a);^9,35^ the strongest defect was present for the p.G433fs construct, used as an additional control.

To test the stability of mutant SMAD6 proteins, we performed western blot analysis of the protein lysates generated for the BRE-luc reporter assay. Strikingly, all 11 CRS-associated rare damaging missense variants (and, in addition, a 5–amino acid in-frame duplication affecting the MH1 domain, and the C-terminal frameshift p.G433fs) exhibited significantly reduced SMAD6 protein levels, suggesting that the substitutions caused protein instability (Fig. 3b, red and pink bars). With the exception of p.E171K, average protein levels were <60% of WT. Conversely, no major instability was observed for eight other novel/rare variants predicted to have lower pathogenicity, or in more frequent (AF_max_ ≥ 0.000045) variants (Fig. 3b, gray bars). As an additional control we generated WT revertants of plasmids encoding three of the missense substitutions classified as pathogenic, and found that the revertant proteins were stable (Fig. S5).

Collectively, aside from the ten frameshifts/stop-gains (likely LoF alleles), we provide compelling functional evidence to support the pathogenicity of all 15 other CRS-associated rare and predicted damaging variants. The damaging missense variants mainly cluster in the MH1/MH2 domains of SMAD6 and significantly affect its activity and/or stability. We observed a strong negative correlation between the predicted deleteriousness of each missense variant and observed protein stability, with the DS (Spearman *r* = −0.67) (Fig. 3c) and CADD (Spearman *r* = −0.66) scores performing similarly (Fig. S6).

### Re-evaluation of *SMAD6* variants previously reported as pathogenic

An enrichment of *SMAD6* variants considered to be pathogenic has been reported in several pathologies distinct from CRS.^10–17^ Using our variant categorization (based on AF and DS), we evaluated all *SMAD6* variants that were previously reported as pathogenic. Systematic review identified 74 different *SMAD6* variants, including 30 missense (Table S4, Fig. S7). Four of the 74 variants (including 3 missense) have an AF_max_ ≥ 0.000045; the in-frame deletion c.79_84del (p.S27_G28del, AF_max_ = 0.0007) is particularly frequent. This variant was identified in our initial resequencing (Table S2) but was excluded based on frequency and occurrence in a poorly conserved region outside the functional domains. Approximately one third of the previously reported SMAD6 missense variants are predicted to have a low (≤3) DS (Table S4, Fig. S8), affecting residues with low evolutionary conservation (Fig. S9).

### Genotyping of *BMP2* polymorphism (rs1884302) in *SMAD6*-positive individuals

Although 3 of the CRS-associated *SMAD6* variants arose de novo, in 20 cases, the variant was transmitted from a clinically unaffected parent (11 mothers, 9 fathers); in 3 further cases, 1 or both parents were not available. The *SMAD6*-transmitting parents had a total of 23 children unaffected by CRS; taking account of the two affected sib pairs in the cohort, this indicates a sib recurrence risk of 2/25 = 8%, equivalent to an estimated penetrance for CRS of *SMAD6* variants of ~16% assuming a 50% transmission rate of the parental *SMAD6* variant (unaffected offspring were not genotyped).

To compare our results with the previous work of Timberlake et al.,^4,18^ which proposed that the rs1884302 SNP influenced the phenotype associated with *SMAD6* variants, we genotyped rs1884302 in all *SMAD6*-positive individuals. However, we did not observe any association in our data between presence of the *BMP2* risk allele (rs1884302C) and manifestation with CRS, whether the data were analyzed in a 2×2 association (Table 2) or by transmission disequilibrium test (in the 20 families with a *SMAD6* carrier parent, parents heterozygous for the rs1884302 SNP transmitted 12 C and 7 T alleles to their affected offspring; *P* = 0.25, χ^2^ test). Merger of our 2×2 association data with the previous results of Timberlake et al.^18^ did however show a significant association (*P* = 0.002, Fisher’s exact test), although this was much weaker than observed in the original publication (Table 2).

**Table 2:** Risk of craniosynostosis in *SMAD6* variant carriers in the presence/absence of the *BMP2* risk allele (C).

	*SMAD6* / *BMP2* genotypes	CRS (+)	CRS (-)	Fisher’s exact *P* value (one-tailed)
This work	*SMAD6* (+) / *BMP2* risk allele (+)	17	12	
	*SMAD6* (+) / *BMP2* risk allele (-)	11	8	0.60
Timberlake et al.^4,18^	*SMAD6* (+) / *BMP2* risk allele (+)	15	1	
	*SMAD6* (+) / *BMP2* risk allele (-)	6	19	0.000011
Combined	*SMAD6* (+) / *BMP2* risk allele (+)	32	13	
	*SMAD6* (+) / *BMP2* risk allele (-)	17	27	0.0019

*CRS* craniosynostosis.

## DISCUSSION

This study builds upon the observations of Timberlake et al.^4,18^ to obtain a more comprehensive picture of the significance and impact of *SMAD6* variants in CRS. We identified 28 affected individuals from 26 different families, adding to the 22 affected individuals from 18 families previously reported.^18^ By surveying all types of CRS without a genetic diagnosis, we gained a broader picture of the range of CRS phenotypes with which *SMAD6* variants may be associated. We find that MS is most highly represented; 5.8% of patients with this diagnosis (Fig. 2a) had a rare, damaging *SMAD6* variant, by far the largest monogenic contribution to MS yet identified.^2^ *SMAD6* variants were less commonly associated with other types of suture fusion, in particular variants were significantly less frequent in SS (0.95% of cases overall) than in MS. We observed four *SMAD6*-positive patients in whom fusion of one or both coronal sutures accompanied the SS, as well as two patients with isolated unicoronal synostosis (Fig. 2b, c). Although based on small numbers, the observation that three subjects developed raised ICP following their primary surgical procedure suggests that long-term postoperative follow-up of these patients is important for optimal management. Given the inhibitory action of SMAD6 on TGFβ/BMP signaling, this work adds further evidence that overactivity of these pathway(s) predispose to craniosynostosis.^1^

Overall, we believe that the data presented here support adoption of *SMAD6* genetic testing to inform genetic diagnosis of CRS: in the 13-year Oxford birth cohort study,^2^ 10 of 677 (1.5%) individuals harbor rare, damaging *SMAD6* variants, making *SMAD6* the fifth most common gene for which variants are found in CRS within this cohort. The pattern of variants identified in patients with CRS (enrichment for heterozygous LoF, plus damaging missense), supports a haploinsufficiency mechanism of pathogenesis, predicting that partial or complete heterozygous deletions of *SMAD6* would also be pathogenic. We screened for such lesions both experimentally (MLPA in 127 individuals with MS) and bioinformatically (113 individuals from 98 families with CRS), but did not detect any deletions in these samples or data sets (Supplementary Information).

The additional observation by Timberlake et al. that the genotype at the rs1884302 SNP appeared strongly related to manifestation of CRS in *SMAD6*-positive individuals^4,18^ has attracted attention as a potential example of digenic or two-locus inheritance.^36^ However, our own data do not support any major modifying role for this SNP (Table 2). Although upon merger with the previous data the overall effect remains significant (Table 2), we note that, given the frequency of the risk allele (C) of ~0.33 (gnomAD, European non-Finnish), the signal in these previous data^4,18^ was largely driven by strong underrepresentation of the C allele in nonpenetrant individuals, with only weak overrepresentation of C in penetrant individuals. This pattern runs counter to population genetic expectations, given the very low penetrance of CRS in individuals heterozygous for pathogenic *SMAD6* variants (formally estimated as 0.16 from the number of additional affected and unaffected offspring born to carrier parents). Furthermore we note that the rs1884302 SNP was originally investigated because it showed the strongest relationship with nonsyndromic SS in a GWAS,^19^ but more recent data for MS reveal no equivalent association for this SNP.^37^ Given that the majority of individuals with deleterious *SMAD6* variants have MS, it is perhaps unsurprising that we did not find an overall interaction between *SMAD6* variants and rs1884302 genotype. Whether such a relationship might exist for the *SMAD6*-positive SS, and whether this interaction could be synergistic or simply represent an additive effect of the GWAS signal, will require a much larger sample size to answer. In conclusion, we caution that the modifying effect of rs1884302 on CRS phenotype in *SMAD6* heterozygotes is unlikely to have useful predictive clinical application.

Despite the compelling statistical relationship between rare, damaging *SMAD6* variation and CRS, many aspects of *SMAD6* disease pathogenesis remain mysterious, a situation that poses substantial challenges for genetic counseling. Notably, although the spectrum of associated pathogenic variants shows the classical pattern of haploinsufficiency, the gnomAD pLI (probability of loss-of-function intolerance) value for *SMAD6* is zero, as the number of LoF variants observed (23) exceeds expectation (11.7).^21^ Our own data support that many individuals carrying *SMAD6* pathogenic variants remain asymptomatic, yet a minority are associated with significant morbidity. Besides the CRS by which these patients were ascertained, in eight cases syndromes were diagnosed based on concurrent congenital cardiac and/or significant neurodevelopmental disorders, features that have been shown to be associated with *SMAD6* variants in independent studies.^10–12,16^

Substantial further work will be required to delineate the overall contribution of *SMAD6* variants to multisystem pathogenesis, and to understand the causal mechanisms underlying the extreme variability in expressivity and the unpredictable penetrance. In this regard, for optimum practice it will be essential carefully to evaluate the pathogenic contribution of every *SMAD6* variant encountered, because it is evident that there are many *SMAD6* variants of intermediate rarity that may be found in mutational screens and assumed to be pathogenic, when this conclusion could be incorrect. Here, we show that in our CRS cohort, by applying joint thresholds for high predicted deleteriousness (DS ≥ 4) and low allele frequency (AF_max_ < 0.000045), we could successfully discriminate missense variants that demonstrated objective evidence of abnormal protein function in assays, based on overall stability (Fig. 3b, c) and (for those located in the MH2 domain), failure to suppress BMP-mediated signaling (Fig. 3a). In our analysis of previously reported missense variants implied to be pathogenic, 10/29 did not meet these joint criteria (Table S4). Such *SMAD6* variants should be prioritized for further functional analysis to enable their clinical significance to be determined robustly, using logic similar to that recently described for *CFTR* variants.^38^

In determining the clinical utility of *SMAD6* genetic testing, prior reports that subjects with BAV and ascending TAA are enriched for *SMAD6* variation are particularly important, because these are occult cardiovascular disorders associated with severe age-related complications, potentially amenable to targeted echocardiographic screening and intervention. At present, however, the absolute and age-related risks of these disorders when association with *SMAD6* variants are unknown. For families within the UK, we have started to offer echocardiographic screening to asymptomatic *SMAD6*-positive parents of children with CRS. Of the seven parents screened to date, none has shown evidence of either BAV or TAA, but unexpectedly, one had severe primary pulmonary hypertension (PPH). While this is not a described association of *SMAD6* variants,^39^ two of the major known monogenic predispositions to PPH (variants in *BMPR2* and *SMAD9*) also involve components of the BMP signaling pathway, so a pathogenic link to *SMAD6* is plausible. These observations should motivate further efforts to disentangle the complex role of *SMAD6* variation in multiple diseases.

## Supplementary information

Supplementary Information

Supplementary Table S1

Supplementary Table S2

Supplementary Table S3

Supplementary Table S4

Genomics England Research Consortium
