# Correction: *SMAD6* variants in craniosynostosis: genotype and phenotype evaluation

**DOI:** 10.1038/s41436-020-0886-2

**Published:** 2020-07-07

**Authors:** Eduardo Calpena, Araceli Cuellar, Krithi Bala, Sigrid M. A. Swagemakers, Nils Koelling, Simon J. McGowan, Julie M. Phipps, Meena Balasubramanian, Michael L. Cunningham, Sofia Douzgou, Wanda Lattanzi, Jenny E. V. Morton, Deborah Shears, Astrid Weber, Louise C. Wilson, Helen Lord, Tracy Lester, David Johnson, Steven A. Wall, Stephen R. F. Twigg, Irene M. J. Mathijssen, Freya Boardman-Pretty, Genomics England Research Consortium, Simeon A. Boyadjiev, Andrew O. M. Wilkie

**Affiliations:** 1 MRCWeatherall Institute of MolecularMedicine, University of Oxford, John Radcliffe Hospital, Oxford, UK; 2 Department of Pediatrics, University of California–Davis, Sacramento, CA USA; 3 Departments of Pathology and Bioinformatics, Erasmus MC, University Medical Center Rotterdam, Rotterdam, The Netherlands; 4 Sheffield Clinical Genetics Service, Sheffield Children’s NHS Foundation Trust, Sheffield, UK; 5 Division of Craniofacial Medicine, Department of Pediatrics, University of Washington, Seattle, WA USA; 6 Manchester Centre for Genomic Medicine, Central Manchester University Hospitals NHS Foundation Trust, Saint Mary’s Hospital, Manchester, UK; 7 Division of Evolution and Genomic Sciences, School of Biological Sciences, Faculty of Biology, Medicines and Health, University of Manchester, Manchester, UK; 8 Fondazione Policlinico Universitario A. Gemelli IRCCS, Rome, Italy; 9 Department of Life Science and Public Health, Università Cattolica del Sacro Cuore, Rome, Italy; 10 West Midlands Regional Clinical Genetics Service and Birmingham Health Partners, Birmingham Women’s and Children’s Hospitals NHS Foundation Trust, Birmingham, UK; 11 Oxford Centre for Genomic Medicine, Oxford University Hospitals NHS Foundation Trust, Oxford, UK; 12 Craniofacial Unit, Oxford University Hospitals NHS Trust, John Radcliffe Hospital, Oxford, UK; 13 Department of Clinical Genetics, Liverpool Women’s NHS Foundation Trust, Liverpool, UK; 14 Clinical Genetics Service, Great Ormond Street Hospital for Children NHS Foundation Trust, London, UK; 15 Oxford Genetics Laboratories, Oxford University Hospitals NHS Foundation Trust, The Churchill Hospital, Oxford, UK; 16 Department of Plastic and Reconstructive Surgery and Hand Surgery, Erasmus MC, University Medical Center Rotterdam, Rotterdam, the Netherlands; 17 Genomics England, London, UK; 18 William Harvey Research Institute, Queen Mary University of London, London, UK

Correction to: *Genetics in Medicine* 2020; 10.1038/s41436-020-0817-2, published online 05 June 2020

The version of the Article previously published did not acknowledge Freya Boardman-Pretty^17,18^ and the Genomics England Research Consortium in the author list. This has now been corrected in both the PDF and HTML versions of the Article.

^17^Genomics England, London, UK; ^18^William Harvey Research Institute, Queen Mary University of London, London, UK

