# Supplementary material for: SMAD6 variants in craniosynostosis: genotype and phenotype evaluation
Source: Genet Med. 2020 Jun 5;22(9):1498–506. doi: 10.1038/s41436-020-0817-2 (PMC7462747; doi:10.1038/s41436-020-0817-2)
Supplement: Supplementary file 1 — Supplementary Information [file 41436_2020_817_MOESM1_ESM.docx]

**Supplementary Information**

**Supplementary Materials and Methods**

**Patients**

The clinical studies were approved by respective Institutional Review Boards (see main text for details). Work on 100,000 Genomes Project (100kGP) samples was performed as part of approved project RR65 in the Research Registry. Written informed consent to obtain samples for genetics research was given by each child’s parent or guardian. Written authorization for publication of clinical photographs was obtained from every individual and/or their parent/guardian. The clinical diagnosis of CRS was confirmed by 3-dimensional computed tomography scanning of the skull. Patients were considered to have a syndromic diagnosis if (i) additional dysmorphic features or congenital anomalies were present and/or (ii) there was significant developmental delay or intellectual disability on neuropsychological assessment; in addition (iii) families including affected first degree relatives were classified as syndromic, although affected individuals in those families may have presented with non-syndromic clinical features. Venous blood was obtained for DNA extraction, and skin was obtained from the site of scalp incision during surgery for CRS. When clinically indicated, samples were tested for mutation hotspots in *FGFR2*, *FGFR3*, *TWIST1*, *TCF12* and *ERF*,^1^ and significant chromosome aneuploidy was investigated using array comparative genomic hybridization; samples with identified mutations known to cause CRS were excluded.

**Resequencing, bioinformatics analysis and variant validation**

Variant screening was performed by next generation sequencing (NGS)-based resequencing of PCR products encompassing the coding regions and intron/exon boundaries of the four exons of *SMAD6* (NC_000015.10 [chr15:66702110-66782849, hg38]; NM_005585, ENST00000288840.9). Universal CS1 (5′-ACACTGACGACATGGTTCTACA-3′) and CS2 (5′-TACGGTAGCAGAGACTTGGTCT-3′) adaptor sequences were included at the 5′ ends of all target-specific forward and reverse primers, respectively (Table S1). Target regions were amplified using multiplexed PCR (eight amplicons in two different mixes, Table S1), from 20 ng of genomic DNA using FastStart Taq DNA Polymerase (Roche) and the following cycling conditions: 8 min denaturation step at 95°C, followed by 35 cycles of 95°C for 30 s, 65°C for 30 s and 72°C for 35 s, and a final extension step of 72°C for 7 min. For each patient, the multiplexed PCR products were combined, diluted 100-fold and used in a second 9-cycle PCR reaction, designed to incorporate Illumina sequence-specific adaptors and sample indexes (Fluidigm) and using the Q5 High-Fidelity DNA Polymerase (NEB). The indexed PCR products were pooled, purified with AMPure XP beads (Beckman Coulter Ltd) and quantified using an Agilent 2200 TapeStation with High Sensitivity D1000 ScreenTape (Agilent Technologies) and the Qubit dsDNA HS Assay Kit (Qubit). Pooled libraries were diluted to a final concentration of 9 pM and sequenced using MiSeq 500 or 600 cycle reagent kits on the MiSeq platform (Illumina). The following custom primers were used for sequencing on the MiSeq: custom CS1-Seq primer for read 1 (5′-A+CA+CTG+ACGACATGGTTCTACA-3′, where + indicates a LNA (Locked Nucleic Acid)-modified nucleotide, CS2-Seq primer for read 2 (5′-T+AC+GGT+AGCAGAGACTTGGTCT-3′) and RC-CS2 primer for the indexing read (5′-A+GAC+CA+AGTCTCTGCTACCGTA-3′). The raw sequencing reads were aligned to the GRCh37/hg19 reference genome. Variant calls and coverage information were obtained using the bioinformatic tool amplimap as previously described.^2,3^

Validation and segregation analysis (using parental samples, if available) of the variants identified by the resequencing were undertaken by dideoxy-sequencing of PCR products from genomic DNA using BigDye Terminator v3.1 (Applied Biosystems). We included additional independently identified *SMAD6* variants (summarized in Table S2) that were detected either by (i) targeted *SMAD6* resequencing of subjects with metopic synostosis collected by the International Craniosynostosis Consortium (variants c.465_471dup, c.715G>A, c.1168G>C) or of subjects with any CRS submitted to the Oxford NHS Genetics Laboratory (variants c.124C>T, c.262G>T, c.817G>C, c.1184A>G, c.1417G>A); or (ii) by genome sequencing of subjects with CRS recruited to the UK’s 100kGP (variants c.40T>C, c.781T>C) or Rotterdam study (variant c.775del). The rs1884302 *BMP2* polymorphism was genotyped in all the *SMAD6*-positive individuals by PCR (Table S1) followed by EcoRI digest and/or dideoxy-sequencing. In Family M130 with two affected children, it was assumed that the *SMAD6* variant was transmitted from the unaffected father (not available for analysis); his rs1884302 genotype could be either CC or CT, so he was considered informative for the presence of the *BMP2* risk allele (Table 2), but not for assessment of transmission disequilibrium.

**Frequency- and deleteriousness-based variant stratification**

To establish frequency-based filtering, as a starting point we used the framework provided by Whiffin *et al*.^4^ The largest proportion of cases attributable to a single nucleotide substitution in CRS is the missense variant *FGFR3* c.749C>G (p.P250R), found in 24/666 CRS cases,^1^ which we use to estimate the maximum expected population allele frequency (AF). Assuming penetrance 0.2, the maximum expected population AF is 1/2000 x 24/666 x 1/2 x 1/0.2 = 0.000045, which would constitute the maximum credible population AF for any causative variant in CRS. Noting that some *SMAD6* variants show marked variation in AF between populations, we assigned AF_max_, the greater of (i) the maximum observed AF in any population in gnomAD^5^ (except Other; gnomAD v2.1.1), where this was based on the presence of ≥2 mutant alleles in that population, or (ii) the overall AF. We classified rare alleles as those for which AF_max_ was < 0.000045.

To measure the deleteriousness of the identified variants, the Deleterious Score (DS, range 0-6) was calculated based on exceeding a defined threshold for six separate criteria.^6^ The DS uses the information provided by six of the scores generated by Annovar.^7^ By default the DS is set to zero, the following predictions all increment the DS by 1: SIFT = 'D', PolyPhen2 = 'D', LRT = 'D', MutationTaster = 'A' or 'D', GERP++ ≥ 5, PhyloP > 1. In addition, the DS is automatically set to 6 if the exonic_func is either a stop-gain, frameshift or splicing. We classified damaging variants as those predicted to affect the functionality (predicted loss-of-function [LoF] and missense variants with DS ≥ 4). For comparison, the CADD score was additionally calculated for missense variants.^8^

For all *SMAD6* variants listed in previous publications^9-18^ and implied to be pathogenic, the nomenclature was verified using Mutalyzer^19^ and their presence and AF checked in gnomAD. We calculated the DS for each variant, and in addition the CADD score for all missense variants.

**Constructs, cell culture and transfection**

We sourced the following plasmids for functional studies: pCMV5-FLAG-SMAD6 containing the human cDNA open reading frame of *SMAD6* with N-terminal FLAG tag (ID 19766, MRC PPU Reagents and Services, University of Dundee, UK); psiCHECK‐2 dual‐luciferase reporter;^20^ pRL-TK containing *Renilla* luciferase reporter; and the empty vector pcDNA3.1. Plasmids pcDNA3-HA-caBMPR1A containing the constitutively active mutant version (p.Q233D) of human BMPR1A with C-terminal HA tag, and pGL3-BRE-luc containing BMP/SMAD responsive elements were kindly provided by Prof. Peter ten Dijke (Leiden University Medical Center, Netherlands).^21^

To analyse the effect of the *SMAD6* c.-9G>T 5′ untranslated region (UTR) variant on protein translation, the *SMAD6* 5′ UTR (~1 kb) was PCR-amplified (primers in Table S1) from the genomic DNA of a carrier individual (Family M130) and cloned into the NheI site of psiCHECK‐2 to generate psiCHECK2-SMAD6-5′UTR-WT and -c.-9G>T constructs. As an additional control, a construct containing the rs559095945 (c.-9G>A) variant was generated by PCR (variant introduced in the reverse primer; Table S1). To analyse *SMAD6* variants affecting the protein-coding region, mutations were generated following the Q5-Mutagenesis kit protocol (New England Biolabs), using primers listed in Table S1 and the pCMV5-FLAG-SMAD6 plasmid as template. All constructs generated were verified by dideoxy-sequencing. Note that the protein predicted by the frameshift construct denoted p.Gly433fs in Fig.3 differs from the similarly annotated human variant, because of sequence differences beyond the location of the normal stop codon (plasmid vector sequence instead of 3' untranslated region).

Fibroblasts, lymphoblastoid cell lines (LCLs) or C2C12 (91031101, Sigma-Aldrich) cells were cultured in DMEM supplemented with L-glutamine, penicillin-streptomycin and either 10% (fibroblasts) or 15% (LCLs and C2C12 cells) fetal bovine serum (FBS), at 37°C under 5% CO_2_. For the functional assays, transfections were performed using Lipofectamine 2000 (Thermo Fisher Scientific) according to the manufacturer’s instructions and cells were processed after 24 h.

**Functional analyses**

For analysis of the c.817G>C and c.817+2T>A splice-site variants, the respective proband’s LCLs or fibroblasts were cultured in parallel with a control individual. Cells were collected and total RNA extracted using TRIzol reagent (Invitrogen), and treated with DNase I (Sigma-Aldrich) to remove genomic DNA. cDNA was prepared using the RevertAid First Strand cDNA Synthesis kit (ThermoFisher Scientific), following the manufacturer’s recommendations for GC-rich regions, and used as template for reverse transcriptase (RT)-PCR (primers in Table S1). Products were visualized by gel electrophoresis; the aberrant product from sample 3711 (c.817+2T>A ) was excised from the agarose gel, extracted using the High Pure PCR Product Purification Kit (Roche) and cloned with the TOPO-TA cloning kit (Thermo Fisher Scientific), whereas the PCR product from sample 8391 (c.817G>C) was directly used for the TOPO-TA cloning step. After colony PCR, positive colonies were randomly selected for dideoxy-sequencing (four from sample 3711 and eight from sample 8391).

To test whether the c.-9G>T 5′ untranslated region (UTR) variant might affect translation efficiency, a dual luciferase reporter analysis was performed as previously described.^20,22^ C2C12 cells were transfected with psiCHECK2-SMAD6-5′UTR-WT, in which the *Renilla* luciferase translation initiated at the *SMAD6* start codon, or with the modified versions containing the c.-9G>T or c.-9G>A variants. After 24h, luciferase activities were measured in lysates using Dual‐Glo Luciferase Reporter Assay System (Promega) to obtain the relative expression of *Renilla* luciferase to the internal Firefly luciferase control. Data were normalized using Firefly luciferase activity and relativized to the WT construct.

To measure SMAD6-mediated inhibition of BMP signalling, dual luciferase assays were performed based on a previously described system,^15^ using a luciferase transcriptional reporter construct containing BMP/SMAD responsive elements (BRE-luc),^21,23^ and utilizing a full-length *SMAD6* construct instead of the shorter isoform previously described.^15^ Using the C2C12 mouse muscle myoblast cell line, BMPs were shown to activate the BRE-luc reporter in a strictly dose-dependent manner.^21^ C2C12 cells seeded in 12‐well plates were transfected with pcDNA3-HA-caBMPR1A (activator of the pathway), pGL3-BRE-luc (reporter) and either wild‐type (WT) or mutant *SMAD6* constructs. The pRL-TK vector was co-transfected to normalize for the transfection levels. Cells were incubated for 24 hr after transfection and luciferase activities were measured in lysates using the Dual‐Glo Luciferase Reporter Assay System (Promega). Data were normalized using *Renilla* luciferase activity and relativized to the WT.

To measure SMAD6 protein stability, aliquots of cell lysates produced in the BRE-luc transcriptional reporter assay were used for standard SDS-PAGE (Mini-Protean TGX 4-15% gradient gels, Bio-Rad) and western blotting (Immobilon-P PVDF membranes, Millipore) procedures. Primary antibodies used were anti-FLAG (F1804, Sigma-Aldrich), anti-HA (3724T, Cell Signaling) and anti-GAPDH (3683, Cell Signaling). For quantitation, captured bands were analysed with Image J software (National Institutes of Health, Bethesda, MD).

**Search for *SMAD6* deletions by MLPA and bioinformatics filtering**

To determine whether partial or complete deletions of *SMAD6* occur frequently in CRS, we used multiplex ligation-dependent probe amplification (MLPA) and bioinformatic filtering, to screen subjects with CRS of unknown cause. For MLPA, synthetic probes (Table S1) were designed according to the manufacturer’s instructions (MRC-Holland) and were used together with control probes as previously described^24,25^ and following the manufacturer’s instructions (MDP version-007; Issued on 01 March 2019; MRC-Holland). MLPA data were analysed with Coffalyser.Net software (MRC-Holland). As a complementary approach we interrogated the Canvas and Manta callsets from Illumina short-read sequencing in the 100kGP (release v7_2019-07-25) for CNV/SV annotations of the *SMAD6* region in 113 undiagnosed subjects (from 98 different families) with CRS.

**Statistical analysis**

Graphs and statistical analyses were conducted with Prism 7 (GraphPad). Luciferase measurements and immunoblot quantifications were performed from at least three independent experiments. *P*-values were calculated using one-way ANOVA with Dunnett’s *post hoc* test, a multiple comparison analysis appropriate for comparing several experimental groups (the different mutant versions) against a single control (the WT construct). Values shown represent means ± standard error of the mean (SEM); in all figures, **P* ≤ 0.05, ***P* ≤ 0.01, ****P* ≤ 0.001.

**Fig. S1. *SMAD6*-positive families identified by NGS-based resequencing.** Filled (gray = non-syndromic, black = syndromic clinical presentation) and unfilled symbols denote individuals with and without craniosynostosis, respectively. Affected sutures are indicated inside the filled symbol (S = sagittal, M = metopic, LC = left coronal, RC = right coronal, BC = bicoronal). Central dots denote *SMAD6* carrier state. The family ID is indicated above each pedigree, together with the *SMAD6* variant. Below each individual analyzed, genotypes are shown for *SMAD6* (with Mut indicating the presence of the damaging allele) and for the *BMP2* polymorphism (rs1884302). The c.-9G>T variant (Family M130) is assumed to have been inherited from the unaffected father, who was unavailable for analysis. In subject 5136 (Family S88), additional bicoronal suture fusion was noted at time of surgery (#).

**Fig. S2. Additional independently identified *SMAD6*-positive CRS patients.** Annotations follow the scheme described in the legend to Fig. S1. NT = not tested.

**Fig. S3. Functional analysis: splice-site variants in *SMAD6*.** **A**, analysis of the c.817G>C splice-site variant identified in Family S410. Amplified cDNA from proband’s (8391) LCL shows abnormal *SMAD6* splice product (indicated by red arrow; black arrow shows the expected size for the amplicon containing the normal splice product), absent in the control (Ctl). Seven of eight randomly selected clones (from TOPO-TA cloning of the RT-PCR product from sample 8391) contained the WT allele (c.817G) with normal splicing (a representative clone A is shown); the remaining clone contained the mutant allele (c.817C) with a 228 nt intronic retention produced by the activation of an intronic cryptic donor splice-site (clone B). **B**, analysis of the c.817+2T>A splice-site variant identified in Family 201. Amplified cDNA from proband’s (3711) fibroblasts shows abnormal *SMAD6* splice products (indicated by red arrow), absent in the control (Ctl). gDNA = genomic DNA negative control. Clone-based sequencing traces show the intronic retention produced by the activation of two different intronic cryptic donor splice-sites. **C**, genomic sequence of *SMAD6* exon 1 (in capitals, with the start codon highlighted in blue) and the 5′ part of intron 1 (lower case). In red is indicated the position of the splice-site variants (c.817G>C and c.817+2T>A) predicted to affect the canonical donor splice-site. Additional predicted (NNSPLICE0.9)^26^ intronic cryptic donor splice-sites are highlighted downstream. The two activated (experimentally demonstrated) intronic cryptic donor splice-sites are boxed in green and purple. **D**, *in silico* translation^27^ of the two abnormally spliced products (both involving partial intron retention) and each generating a frameshift and premature stop codon.

**Fig S4. Functional analysis: 5′ UTR variant in *SMAD6*.** Left, pedigree of Family M130. The c.-9G>T variant is assumed to have been inherited from the unaffected father, who was unavailable for analysis. Middle, the diagram illustrates the positions of the c.-9G>T (in red, creating an out-of-frame upstream ATG) and c.-9G>A (dbSNP rs559095945; in green) variants, relative to the normal ATG initiator codon (blue box). Right, the plot shows the results of the luciferase analysis to determine the effect of the 5′ UTR variants on translation. C2C12 cells were transfected with psiCHECK2-SMAD6-5′UTR-WT (WT), in which the *Renilla* luciferase translation initiated at the *SMAD6* start codon, or with the modified versions containing the c.-9G>T (red bar) or c.-9G>A (green bar) variants. Luciferase activities were measured in lysates to obtain the relative expression of *Renilla* luciferase to the internal Firefly luciferase control. Data were normalized using Firefly luciferase activity and relativized to the WT construct. Plots are shown as means ± SEM based on three independent experiments.

**Fig. S5. Analysis of SMAD6 revertants.** Mutant plasmids engineered to encode three different missense variants (affecting different regions of SMAD6) were selected for reversion to wild type by site-directed mutagenesis; expressed SMAD6 protein levels of the revertants was measured by western blotting. The immunoblot shows SMAD6 protein levels (anti FLAG antibody) and the GAPDH loading control. Controls (negative in green, [p.A325T], positive in blue [p.C484F]) were selected as previously described.^15^ The selected missense variants are colored in red (or in blue, in case of the p.C484F) and their respective revertants are in gray.

**Fig. S6. Correlation between predicted deleteriousness and observed SMAD6 protein levels.** The experimentally determined average protein levels (*y*-axis) are plotted against the predicted deleterious scores for all the functionally assayed missense variants (*x*-axis), using either the DS statistic (top) or CADD score (bottom).

**Fig. S7. Previously reported SMAD6 missense variants.** The positions of previously reported variants (listed at bottom) are shown in comparison with those presented in this work (as displayed in Fig. 1).

**Fig. S8. Deleteriousness evaluation of previously reported SMAD6 missense variants.** Predicted deleterious scores (DS and CADD) are plotted for all published SMAD6 missense variants. Variants with an AF_max_ ≥ 0.000045 are colored in turquoise.

**Fig. S9. Multiple-protein sequence alignment of vertebrate members of SMAD6 and human SMAD7**, indicating the affected amino acids of the pathogenic missense variants identified in this work (red) and the previously reported missense variants (highlighted in yellow). An asterisk (*) indicates positions which have a single, fully conserved residue, a colon (:) indicates conservation between groups of strongly similar properties, and a period (.) indicates conservation between groups of weakly similar properties.

**Supplementary References**

1. Wilkie AOM, Johnson D, Wall SA. Clinical genetics of craniosynostosis. *Curr Opin Pediatr.* 2017;29:622-628.

2. Koelling N, Bernkopf M, Calpena E, et al. amplimap: a versatile tool to process and analyze targeted NGS data. *Bioinformatics.* 2019;35:5349-5350.

3. Reijnders MRF, Miller KA, Alvi M, et al. De novo and inherited loss-of-function variants in TLK2: Clinical and genotype-phenotype evaluation of a distinct neurodevelopmental disorder. *Am J Hum Genet.* 2018;102:1195-1203.

4. Whiffin N, Minikel E, Walsh R, et al. Using high-resolution variant frequencies to empower clinical genome interpretation. *Genet Med.* 2017;19:1151-1158.

5. Lek M, Karczewski KJ, Minikel EV, et al. Analysis of protein-coding genetic variation in 60,706 humans. *Nature.* 2016;536:285-291.

6. Fu W, O'Connor TD, Jun G, et al. Analysis of 6,515 exomes reveals the recent origin of most human protein-coding variants. *Nature.* 2013;493:216-220.

7. Wang K, Li M, Hakonarson H. ANNOVAR: functional annotation of genetic variants from high-throughput sequencing data. *Nucleic Acids Res.* 2010;38:e164.

8. Kircher M, Witten DM, Jain P, O'Roak BJ, Cooper GM, Shendure J. A general framework for estimating the relative pathogenicity of human genetic variants. *Nat Genet.* 2014;46:310-315.

9. Gillis E, Kumar AA, Luyckx I, et al. Candidate Gene Resequencing in a Large Bicuspid Aortic Valve-Associated Thoracic Aortic Aneurysm Cohort: SMAD6 as an Important Contributor. *Front Physiol.* 2017;8:400.

10. Jin SC, Homsy J, Zaidi S, et al. Contribution of rare inherited and de novo variants in 2,871 congenital heart disease probands. *Nat Genet.* 2017;49:1593-1601.

11. Kloth K, Bierhals T, Johannsen J, et al. Biallelic variants in SMAD6 are associated with a complex cardiovascular phenotype. *Hum Genet.* 2019;138:625-634.

12. Lelieveld SH, Reijnders MR, Pfundt R, et al. Meta-analysis of 2,104 trios provides support for 10 new genes for intellectual disability. *Nat Neurosci.* 2016;19:1194-1196.

13. Luyckx I, MacCarrick G, Kempers M, et al. Confirmation of the role of pathogenic SMAD6 variants in bicuspid aortic valve-related aortopathy. *Eur J Hum Genet.* 2019;27:1044-1053.

14. Park JE, Park JS, Jang SY, et al. A novel SMAD6 variant in a patient with severely calcified bicuspid aortic valve and thoracic aortic aneurysm. *Mol Genet Genomic Med.* 2019;7:e620.

15. Tan HL, Glen E, Topf A, et al. Nonsynonymous variants in the SMAD6 gene predispose to congenital cardiovascular malformation. *Hum Mutat.* 2012;33:720-727.

16. Timberlake AT, Choi J, Zaidi S, et al. Two locus inheritance of non-syndromic midline craniosynostosis via rare SMAD6 and common BMP2 alleles. *Elife.* 2016;5:e20125.

17. Timberlake AT, Furey CG, Choi J, et al. De novo mutations in inhibitors of Wnt, BMP, and Ras/ERK signaling pathways in non-syndromic midline craniosynostosis. *Proc Natl Acad Sci USA.* 2017;114:E7341-E7347.

18. Yang Y, Zheng Y, Li W, et al. SMAD6 is frequently mutated in nonsyndromic radioulnar synostosis. *Genet Med.* 2019;21:2577-2585.

19. Wildeman M, van Ophuizen E, den Dunnen JT, Taschner PE. Improving sequence variant descriptions in mutation databases and literature using the Mutalyzer sequence variation nomenclature checker. *Hum Mutat.* 2008;29:6-13.

20. Calvo SE, Pagliarini DJ, Mootha VK. Upstream open reading frames cause widespread reduction of protein expression and are polymorphic among humans. *Proc Natl Acad Sci U S A.* 2009;106:7507-7512.

21. Korchynskyi O, ten Dijke P. Identification and functional characterization of distinct critically important bone morphogenetic protein-specific response elements in the Id1 promoter. *J Biol Chem.* 2002;277:4883-4891.

22. Zhou Y, Koelling N, Fenwick AL, et al. Disruption of TWIST1 translation by 5' UTR variants in Saethre-Chotzen syndrome. *Hum Mutat.* 2018;39:1360-1365.

23. Goto K, Kamiya Y, Imamura T, Miyazono K, Miyazawa K. Selective inhibitory effects of Smad6 on bone morphogenetic protein type I receptors. *J Biol Chem.* 2007;282:20603-20611.

24. Twigg SRF, Vorgia E, McGowan SJ, et al. Reduced dosage of ERF causes complex craniosynostosis in humans and mice and links ERK1/2 signaling to regulation of osteogenesis. *Nat Genet.* 2013;45:308-313.

25. Twigg SRF, Forecki J, Goos JA, et al. Gain-of-function mutations in ZIC1 are associated with coronal craniosynostosis and learning disability. *Am J Hum Genet.* 2015;97:378-388.

26. Reese MG, Eeckman FH, Kulp D, Haussler D. Improved splice site detection in Genie. *J Comput Biol.* 1997;4:311-323.

27. Gasteiger E, Gattiker A, Hoogland C, Ivanyi I, Appel RD, Bairoch A. ExPASy: The proteomics server for in-depth protein knowledge and analysis. *Nucleic Acids Res.* 2003;31:3784-3788.
