# Supplementary material for: SMAD6 variants in craniosynostosis: genotype and phenotype evaluation
Source: Genet Med. 2020 Jun 5;22(9):1498–506. doi: 10.1038/s41436-020-0817-2 (PMC7462747; doi:10.1038/s41436-020-0817-2)
Supplement: Supplementary file 6 — Genomics England Research Consortium [file 41436_2020_817_MOESM6_ESM.docx]

**Genomics England Research Consortium**

J. C. Ambrose^1^, M. Bleda^1^, F. Boardman-Pretty^1, 2^, J. M. Boissiere^1^, C. R. Boustred^1^, M. J. Caulfield^1, 2^, G. C. Chan^1^, C. E. H. Craig^1^, L. C. Daugherty^1^, A. de Burca^1^, A. Devereau^1^, G. Elgar^1, 2^, R. E. Foulger^1^, T. Fowler^1^, P. Furió-Tarí^1^, J. M. Hackett^1^, D. Halai^1^, J. E. Holman^1^, T. J. P. Hubbard^1^, D. Kasperaviciute^1, 2^, M. Kayikci^1^, L. Lahnstein^1^, K. Lawson^1^, S. E. A. Leigh^1^, I. U. S. Leong^1^, F. J. Lopez^1^, F. Maleady-Crowe^1^, J. Mason^1^, E. M. McDonagh^1, 2^, L. Moutsianas^1, 2^, M. Mueller^1, 2^, A. C. Need^1, 2^, C. A. Odhams^1^, C. Patch^1^,2, D. Perez-Gil^1^, D. Polychronopoulos^1^, J. Pullinger^1^, T. Rahim^1^, A. Rendon^1^, T. Rogers^1^, M. Ryten^1^, K. Savage^1^,R. H. Scott^1^, A. Siddiq^1^, A. Sieghart^1^, D. Smedley^1, 2^, K. R. Smith^1, 2^, A. Sosinsky^1, 2^, W. Spooner^1^,H. E. Stevens^1^, A. Stuckey^1^, E. R. A. Thomas^1, 2^, S. R. Thompson^1^, C. Tregidgo^1^, A. Tucci^1, 2^, E. Walsh^1^, S. A. Watters^1^, M. J. Welland^1^, E. Williams^1^, K. Witkowska^1, 2^, S. M. Wood^1, 2^ and M. Zarowiecki^1^

^1^Genomics England, London, UK; ^2^William Harvey Research Institute, Queen Mary University of London, London, UK.
